# Water-Soluble Trichogin GA IV-Derived Peptaibols Protect Tomato Plants From *Botrytis cinerea* Infection With Limited Impact on Plant Defenses

**DOI:** 10.3389/fpls.2022.881961

**Published:** 2022-05-19

**Authors:** Ivan Baccelli, Simone Luti, Rodolfo Bernardi, Francesco Favaron, Marta De Zotti, Luca Sella

**Affiliations:** ^1^Institute for Sustainable Plant Protection, National Research Council of Italy, Sesto Fiorentino, Italy; ^2^Department of Agriculture, Food and Environment, University of Pisa, Pisa, Italy; ^3^Department of Land, Environment, Agriculture, and Forestry (TESAF), University of Padova, Legnaro, Italy; ^4^Department of Chemistry, University of Padova, Padova, Italy

**Keywords:** *Trichoderma*, antimicrobial peptides, peptaibiotics, *Solanum lycopersicum*, Micro-Tom, Marmande, ROS, plant pathogen

## Abstract

Peptaibols are non-ribosomal linear peptides naturally produced by a wide variety of fungi and represent the largest group of peptaibiotic molecules produced by *Trichoderma* species. Trichogin GA IV is an 11-residue *lipo*peptaibol naturally produced by *Trichoderma longibrachiatum*. Peptaibols possess the ability to form pores in lipid membranes or perturb their surface, and have been studied as antibiotics or anticancer drugs in human medicine, or as antimicrobial molecules against plant pathogens. When applied to plants, peptaibols may also elicit defense responses. A major drawback to the exploitation and application of peptaibols in agriculture is their poor water solubility. In a previous study, we designed water-soluble Lys-containing Trichogin GA IV analogs, which were able to inhibit the growth of several fungal plant pathogens *in vitro*. In the present study, we shed light on the mechanism underpinning their efficacy on plants, focusing on six Trichogin GA IV analogs. Our results highlighted peptide hydrophilicity, rather than helix stability, as the major determinant of their activity against *B. cinerea* infection in tomato leaves. The peptides showed preventive but not curative efficacy against infection, and lack of translaminar activity, with results reproducible on two tomato cultivars, Marmande and Micro-Tom. Reactive oxygen species (ROS) detection analysis in tomato and *Arabidopsis*, and expression of defense genes in tomato, highlighted a transient and limited impact of the peptides on the plant defense system. The treatment did not result in significant modulation of defense genes or defense priming. The antimicrobial effect thus emerges as the only mechanism behind the plant protection ability exerted by water-soluble Trichogin GA IV analogs, and limited effects on the plant metabolism are expected to occur.

## Introduction

Peptaibols are a class of non-ribosomal linear peptides naturally produced by a variety of fungi, mostly belonging to the order Hypocreales, such as *Clonostachys* spp., *Mycogone* spp., and *Sepedonium* spp. among others, although *Trichoderma* species seem actually to be the main producers (Szekeres et al., 2005; Leitgeb et al., 2007; Montesinos, 2007). The first peptaibols were isolated in the late 1960s from *Trichoderma viride* and named suzukacillin and alamethicin (Leitgeb et al., 2007). Peptaibols are 5–20 residue-long peptides characterized by the presence of an acylated N terminus, non-proteinogenic amino acids, such as isovaline (Iva) or α-aminoisobutyric acid (Aib), and a C-terminal amino alcohol. Accordingly, their name derives from peptide + Aib + amino alcohol (pept-aib-ol; Szekeres et al., 2005; Leitgeb et al., 2007). Peptaibols represent the largest group of peptaibiotics produced by *Trichoderma* species (Zeilinger et al., 2016).

Peptaibols possess stable and amphipathic helical structures and can self-assemble to form pores in lipid membranes or perturb their surface (Szekeres et al., 2005; Zeilinger et al., 2016). The membrane activity of these metabolites has led several researchers to investigate their possible exploitation as antibiotics or anticancer drugs in human medicine (Leitgeb et al., 2007; Shi et al., 2010; Dalzini et al., 2016). Peptaibols possess interesting features also for plant protection purposes. Indeed, they have been found to act as antimicrobial molecules against plant pathogens (Shi et al., 2012; Zeilinger et al., 2016; De Zotti et al., 2020; Sella et al., 2021) and to elicit defense reactions in various plants (Engelberth et al., 2000; Viterbo et al., 2007; Luo et al., 2010; Rippa et al., 2010). It is not clear whether peptaibols may act as defense priming stimuli (Mauch-Mani et al., 2017; Baccelli et al., 2020).

As natural peptide molecules, peptaibols are expected to degrade to nontoxic amino acids when introduced into the environment, thus meeting current legislation requirements and public acceptance (De Zotti et al., 2020). Various limitations however hamper their exploitation in agriculture, in particular, difficulties regarding the purification procedures from naturally-producing fungi and, above all, their poor solubility in water (De Zotti et al., 2020). For these reasons, our group recently designed water-soluble analogs of the *lipo*peptaibol Trichogin GA IV for plant protection purposes (De Zotti et al., 2020; Sella et al., 2021).

Trichogin GA IV is a short-length (11 residues) helical and hydrophobic *lipo*peptaibol naturally produced by the fungus *T. longibrachiatum* (Rebuffat et al., 1999; Toniolo et al., 2001a). Native Trichogin GA IV possesses three Aib residues within its sequence, it is blocked at its N-terminus by an *n*-octanoyl (*n*-Oct) moiety and has a 1,2-aminoalcohol (Leucinol, Lol) at its C-terminus (De Zotti et al., 2012a). Trichogin GA IV shows remarkable resistance to proteolysis mostly due to its well-developed helical conformation and possesses antibacterial activity against methicillin-resistant human pathogens (De Zotti et al., 2009). We recently reported rationally-designed, water-soluble, Lys-containing Trichogin GA IV analogs with antimicrobial activity against fungal plant pathogens (De Zotti et al., 2020; Sella et al., 2021). In particular, Pep 4 and Pep 4r, both sharing Gly to Lys substitutions at positions 5 and 6 (K5K6), with Pep 4r also bearing a Lol to Leu-NH_2_ substitution at its C-terminus, inhibited *in vitro* growth of the fungal plant pathogens *Botrytis cinerea*, *Bipolaris sorokiniana*, *Fusarium graminearum*, *Penicillium expansum*, and *Pyricularia oryzae*. In addition, they were effective in preventing *B. cinerea* infections on grape and bean tissues, and *P. oryzae* infections on barley and rice leaves, without apparent phytotoxic effects when sprayed at a concentration of 50 μM (De Zotti et al., 2020; Sella et al., 2021).

In the present study, we investigate the mechanism behind their efficacy in planta, by focusing on the protection against *B. cinerea* infection in tomato. To this purpose, we used six Trichogin GA IV-derived peptides bearing Gly to Lys, Lol to Leu-NH_2_, and/or Gly to Aib substitutions, two of which designed and produced in the present study (Table 1). Reactive oxygen species (ROS) induction on tomato and *Arabidopsis* leaves, and expression of defense genes in tomato were studied to elucidate the ability of the peptides to induce, or prime, plant defenses.

**Table 1:** Sequence and chemical features of Trichogin GA IV-derived peptides tested in the present study.

Name	Sequence^a^	Helix stability^b^	Hydrophilicity^b^^,^^c^
Trichogin GA IV	1-octanoyl-Aib-Gly-Leu-Aib-Gly-Gly-Leu-Aib-Gly-Ile-Lol	Native	Native
Pep 4^d^	1-octanoyl-Aib-Gly-Leu-Aib-**Lys**-**Lys**-Leu-Aib-Gly-Ile-Lol	+	++
Pep 4r^d^	1-octanoyl-Aib-Gly-Leu-Aib-**Lys**-**Lys**-Leu-Aib-Gly-Ile-**Leu-NH**_**2**_	++	++
Pep K9	1-octanoyl-Aib-Gly-Leu-Aib-Gly-Gly-Leu-Aib-**Lys**-Ile-Lol	=	+
Pep 5^d^	1-octanoyl-Aib-Gly-Leu-Aib-Gly-**Lys**-Leu-Aib-Gly-Ile-Lol	=	+
Pep 6^d^	1-octanoyl-Aib-Gly-Leu-Aib-**Lys**-**Aib**-Leu-Aib-Gly-Ile-Lol	++	+
Pep 6r	1-octanoyl-Aib-Gly-Leu-Aib-**Lys**-**Aib**-Leu-Aib-Gly-Ile-**Leu-NH**_**2**_	+++	+

Modifications to the native sequence are highlighted in bold.

a Aib, α-aminoisobutyric acid; Lol, leucinol.

b Comparisons to the native peptide Trichogin GA IV (+, more stability; =, similar stability).

c As evaluated from their respective HPLC retention times.

d Peptides designed in De Zotti et al. (2020).

## Materials and Methods

### Peptide Design and Synthesis

Trichogin GA IV-derived peptides (Table 1) were produced by solid-phase peptide synthesis as described in De Zotti et al. (2020). The C-terminal Leucinol was obtained by using a 2-chlorotrytil resin preloaded with the 1,2-amino alcohol. A yield of about 70% on the purified peptides (purity > 95%) was achieved for all analogs. Pep K9 and Pep 6r were designed and synthesized in the present study. Details on the design and synthesis of Pep 4, Pep 4r, Pep 5, and Pep 6 are provided in De Zotti et al. (2020). C-terminal amide peptides were produced on a Rink-amide resin. Purity was checked by analytical HPLC on a C18 column (Phenomenex Jupiter 5 μm, 300 Å, 250 × 4.5 mm). The gradient used was 60%–100% B in 30 min; eluent A H_2_O/CH_3_CN 9:1 with 0.05% trifluoroacetic acid (TFA); eluent B CH_3_CN/H_2_O 9:1 with 0.05% TFA. HPLC retention times were used to evaluate hydrophilicity. Helix stability was evaluated by combining literature computational considerations (Chou and Fasman, 1978; Lyu et al., 1991), and circular dichroism analysis performed on Trichogin analogs in solvents of different polarity (De Zotti et al., 2012a).

### Plant Material and Growth Conditions

Tomato (*Solanum lycopersicum*) seeds belonging to the cultivars Marmande and Micro-Tom were purchased from two specialty stores: Cooperativa Agricola Legnaia (Florence, Italy) and Gargini Sementi (Lucca, Italy), respectively. Plants were sown in Jiffy-7 peat pellets and transferred into commercial soil 2 weeks after germination. Plants were grown on a bench in a growth room under LED lights “Toplight Greenhouse Plus—Natural” (35% Blue, 17% Green; and 48% Red; C-LED srl, Imola, Italy) with 12 h of light (650 μmol m^2^ s^−1^), at 28°C (day)/21°C (night), until treatment.

For ROS detection, *Arabidopsis thaliana* ecotype Columbia-0 (Col-0) plants were grown in Jiffy-7 peat pellets in a dedicated growth chamber at 21°C (day)/18°C (night), 12 h of light (100 μmol m^2^ s^−1^), for 5 weeks.

### Pathogen Strain and Cultivation

*Botrytis cinerea* strain B05.10 (Staats and van Kan, 2012) was grown on potato dextrose agar (PDA for microbiology, VWR International srl, Italy) at 21°C (day)/18°C (night), with 12 h of light (100 μmol m^2^ s^−1^) to stimulate and standardize conidia production. These conditions were obtained in the growth chamber used for *A. thaliana* growth, where detached tomato leaves and whole plantlets were also incubated after peptide treatment and during infections. *Botrytis cinerea* cultures were maintained under these conditions until conidia collection generally obtained after 10–15 days.

### Plant Treatments

Treatments with Trichogin GA IV-derived peptides were performed on either detached leaves or whole plantlets, as described below. Peptide treatments for ROS detection were performed as described in the appropriate paragraph.

To test the efficacy of the peptides in preventing *B. cinerea* infection, 10–12 mature and healthy leaves were cut from different Micro-Tom or Marmande plants and placed in 90 mm-Petri dishes containing a filter paper disc wet with 1 ml sterile water, to ensure high internal relative humidity (RH) conditions during incubation. Leaves were sprayed with a 10-ml pump atomizer on the lower (abaxial) leaf surface with 50 μM peptide solution (approximately 300 μl per leaf), or sterile distilled water as a control. Peptides at the concentration of 100 μM were tested only on cv. Marmande leaves, according to the same procedure. In most cases, peptides were sprayed as peptide solution in water, without any addition of adjuvant. However, the use of the commercial surfactant Silwet L-77 AG (Momentive Performance Materials Inc., NY, United States) was assessed in some tests, as specified below and in the result section, with the aim of enhancing the biological activity of the peptides. The plates were sealed with parafilm and incubated in the growth chamber at 21°C (day)/18°C (night), with 12 h of light (100 μmol m^2^ s^−1^) until pathogen inoculation. Inoculations were performed 48 or 120 h after treatment, as described in the next paragraph.

Tests for curative efficacy, i.e., the ability of the peptides to stop ongoing infections, were performed by spraying detached leaves with 50 μM peptides 24 h after *B. cinerea* inoculation (see next paragraph). In this case, Micro-Tom or Marmande infected leaves were sprayed with a peptide solution containing 0.01% v/v Silwet L-77 AG.

To demonstrate the efficacy of Pep 4 to prevent *B. cinerea* infection on whole tomato plants, six 2-week-old Micro-Tom plants were sprayed on the adaxial leaf surfaces with 50 μM Pep 4 (approximately 700 μl per plant) containing 0.01% *v*/*v* Silwet L-77 AG. Six control plants were sprayed with sterile distilled water containing 0.01% *v*/*v* Silwet L-77 AG. After treatment, plants were incubated for 48 h at 21°C (day)/18°C (night), 12 h of light (100 μmol m^2^ s^−1^) until infection.

For gene expression studies, 30 2-week-old Micro-Tom plants were used to investigate the ability of the peptide to induce or prime defense gene expression against *B. cinerea*. In this case, 15 plants were sprayed with 50 μM Pep 4 containing 0.01% v/v Silwet L-77 AG, and 15 plants were sprayed with water containing 0.01% v/v Silwet L-77 AG as a control. Plants were incubated for 48 h as described above, then three control and three Pep 4-treated plants were sampled for RNA extraction (see appropriate paragraph). The remaining plants were inoculated (see next paragraph) or mock treated with potato dextrose broth (PDB, Laboratorios Conda S.A., Spain) yielding the following samples for gene expression studies: control + mock at 6 and 24 h post infection (hpi; i.e., treated with water 48 h before inoculation and then inoculated with PDB and collected at 6 or 24 hpi); control + *B. cinerea* at 6 and 24 hpi (i.e., treated with water 48 h before inoculation and then inoculated with *B. cinerea* and collected at 6 or 24 hpi); Pep 4 + mock at 6 and 24 hpi (i.e., treated with Pep 48 h before inoculation and then inoculated with PDB and collected at 6 or 24 hpi); Pep 4 + *B. cinerea* at 6 and 24 hpi (i.e., treated with Pep 48 h before inoculation and then inoculated with *B. cinerea* and collected at 6 or 24 hpi). At the end of each sampling time, all true leaves from each plant were frozen in liquid nitrogen and stored at −80°C until RNA extraction.

### Pathogen Inoculation and Disease Quantification

All treatments and inoculations in this study were performed 2–3 h after lights in the growth chamber were on. Inoculations on detached leaves were performed by applying a single 10-μl droplet of *B. cinerea* B05.10 conidial suspension (1 × 10^6^ conidia/ml in PDB) on a side of the midrib. In a few cases, bigger leaves were inoculated with two droplets applied on each side of the midrib. Inoculations were performed 48 h after peptide treatment either on the previously treated leaf surface or on the opposite untreated surface, in order to highlight translaminar or resistance-inducing activity (see Results). Inoculations 120 h after treatment were also performed on Pep 4- and Pep 4r-treated leaves in order to assess the duration of the protection. For curative efficacy, untreated healthy leaves were inoculated on the abaxial leaf surface 24 h before peptide treatment. Infected leaves were sealed in Petri dishes containing 1 ml water as described above to maintain high RH conditions needed for infection development, and incubated at 21°C (day)/18°C (night), 12 h of light (100 μmol m^2^ s^−1^). Disease severity was determined 3 days after infection by measuring the necrotic lesion diameter.

Two-week-old Micro-Tom plants were inoculated by spraying adaxial leaf surfaces with a conidial suspension containing 1 × 10^6^ conidia/ml in PDB. Approximately, 800 μl of conidial suspension were sprayed on each plant. For gene expression studies, that amount was decreased to approximately 600 μl per plant, while mock plants were sprayed with the same amount of PDB. Plants were incubated at 21°C (day)/18°C (night), with 12 h of light (100 μmol m^2^ s^−1^), in closed transparent boxes containing free water on the bottom to ensure high RH conditions needed for infection development. Disease severity was determined after 3 days by classifying each plant on the basis of the visible leaf necrosis, as follows: Class I: no or point-limited necrosis; Class II: spreading lesions affecting <50% of the leaf surface; Class III: spreading lesions affecting >50% of the leaf surface; and Class IV: extensive necrosis and plant collapse.

Concerning the plants used for gene expression studies, disease severity was determined at the end of the experiment (24 hpi) by counting the number of necrotic spots visible on the leaf surface.

### RNA Extraction and Gene Expression Analyses

Total RNA was extracted from 80 to 100 mg of ground leaf material by using RNeasy Plant Mini Kit (Qiagen, Italy), with guanidine thiocyanate extraction buffer RLT. RNA was quantified in a Qubit fluorometer (Thermo Fisher Scientific, MA, United States) by using Qubit RNA BR Assay Kit (Life Technologies, Italy). RNA integrity was evaluated by agarose gel electrophoresis. RNA samples (400 ng each) were treated with Amplification Grade DNase I (Sigma-Aldrich) and reverse-transcribed into cDNA by using Maxima First Strand cDNA Synthesis Kit for RT-qPCR (Thermo Fisher Scientific, MA, United States). qPCRs were performed in a StepOne Real-Time PCR System (Applied Biosystems). Reactions (20 μl) were carried out with 20 ng of cDNA, 250 nM of each primer, and Fast SYBR Green Master Mix (Applied Biosystems) by setting the recommended thermal-cycling conditions for the fast mode (Fast SYBR® Green Master Mix Protocol, Applied Biosystems). The following genes were analyzed: *Proteinase inhibitor II* (*PIN2*), *Beta-1,3-glucanase A* (*GluA*), *Pathogenesis-related protein 1* (*PR1*), *1-aminocyclopropane-1-carboxylate oxidase 1* (*ACO1*), *Pathogenesis-related genes transcriptional activator* (*PTI5*), *Lipoxygenase A* (*Lox1.1*), and *Polygalacturonase inhibitor protein* (*PGIP*). Relative gene expression values were calculated by 2^-ΔΔCt^ method as described in Livak and Schmittgen (2001). *Actin-7* was selected as the endogenous reference gene after comparing its transcriptional stability against three candidate reference genes: *Ubiquitin*, *Tubulin*, and *Glyceraldehyde-3-phosphate dehydrogenase* (*GPDH*; Supplementary Figure S1). Transcriptional stability was tested in 10 cDNA samples representative of all the experimental conditions analyzed. Before performing relative expression calculations, the performance of each amplification run was checked by melting curve analysis, and the amplification plots were compared to ensure that amplification efficiencies between target and reference genes were approximately equal (data not shown; Schmittgen and Livak, 2008). The list of genes analyzed in this study and their primer sequences are provided in Supplementary Table S1. The primer sequences were designed in the present study, with the exception of *ACO1*, *Actin-7*, *Ubiquitin*, and *GPDH* (Mannucci et al., 2022).

### Reactive Oxygen Species Detection on Leaves

Trichogin GA IV-derived peptides at the concentration of 50 μM in water were applied as 10-μl droplets on the abaxial surface of detached Micro-Tom and *A. thaliana* leaves. Five droplets were applied on each leaf, and three leaves per treatment were analyzed. Incubations were performed in Petri dishes for 24 and 48 h as previously described. ROS production by leaves was visualized by staining with the cell-permeable probe 2′,7′-dichlorodihydrofluorescein diacetate (H_2_DCFDA; Sigma-Aldrich, United States), as reported in Luti et al. (2020). For staining, leaves were soaked for 1 h in a 10 μM H_2_DCFDA solution in 20 mM phosphate buffer, pH 6.8. After H_2_DCFDA incubation, leaves were washed twice with phosphate buffer and mounted on glass slides for microscopy analysis, which was performed under a confocal Leica TCS SP5 scanning microscope (Leica, Germany; λex 460 and λem 512 nm).

### Statistical Analyses

The efficacy of different peptide treatments against *B. cinerea* infection on detached leaves was analyzed by one-way ANOVA and Tukey–Kramer multiple comparison post-test when *p* < 0.05. Efficacies between two peptide concentrations, or between control and treated leaves, were analyzed by unpaired *t*-test (*p* < 0.05). Gene expression data were analyzed by unpaired *t*-test by comparing to the appropriate calibrator sample (control 48 hpt or control + mock 6 and 24 hpi) as detailed in the figure legends (*p* < 0.05). Analyses were performed in GraphPad software (GraphPad Software Inc., CA, United States). Disease severity was evaluated by Fisher’s exact test (*p* < 0.05).

## Results

### Peptide Design and Characterization

Following a published protocol (De Zotti et al., 2020), we synthesized six Trichogin GA IV-derived peptides with improved water solubility and/or conformational stability (Table 1). The analogs were characterized by one to three amino acid substitutions in the native Trichogin GA IV sequence. Gly to Lys substitutions were performed, with positional differences, in all peptide variants to confer water solubility (Table 1). The Gly-Gly dipeptide in the middle of the native sequence of Trichogin GA IV forms a kink in the 3D-structure, which lessens its helical content (Venanzi et al., 2009). Accordingly, being a better helix promoter than Gly (Chou and Fasman, 1978), Lys was also able to stabilize the peptide structure when placed in that kink. Similarly, the Gly-to-Aib substitution in that kink (Pep 6 and Pep 6r) further rigidified the helix, since Aib is a strong helix-inducer (Toniolo et al., 2001b). Finally, by replacing Gly with Lys at position 9 (Pep K9), i.e., far from that kink, we aimed to selectively evaluate the enhancement of hydrophilicity against helix stabilization effects shown by the other variants (position 9 is already involved in a strong helical segment, see Toniolo et al., 1994). The C-terminal leucine amide in the two most helical peptide analogs (Pep 4r and Pep 6r) introduced an additional hydrogen bond, further strengthening the helix (Table 1).

### Protection by Trichogin GA IV-Derived Peptides Against *Botrytis cinerea* Infection on Tomato Leaves

**Figure 1:**
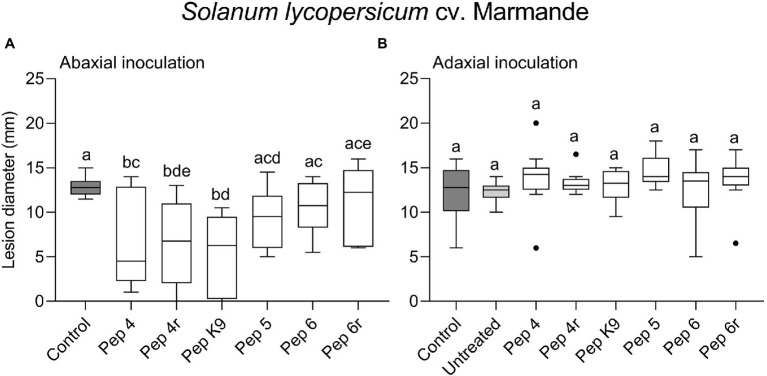
Preventive efficacy of Trichogin GA IV-derived peptides against *Botrytis cinerea* infection on *Solanum lycopersicum* cv. Marmande leaves. Treatments were performed on the lower (abaxial) surface by spraying 50 μM peptide solution in water **(A)**, or in 0.01% v/v of surfactant Silwet L-77 AG **(B)**. The pathogen was inoculated 48 h later by drop inoculation with a conidial suspension of *B. cinerea*. Conidia were applied on the abaxial (peptide-treated; **A**) or adaxial (untreated; **B**) leaf surface. Lesion diameters were measured 3 days after infection. Control leaves were treated with water **(A)** or water containing 0.01% Silwet L-77 **(B)**. Values from 12 biological replicates **(A)** or 9–14 biological replicates **(B)** are reported as Tukey boxplots. Data were analyzed by one-way ANOVA with Tukey–Kramer post-test (*p* < 0.05). Statistically significant differences are shown by different letters.

The efficacy of the six Trichogin-derived peptides (Pep 4, Pep 4r, Pep K9, Pep 5, Pep 6, and Pep 6r, Table 1) against *B. cinerea* infections on tomato was firstly tested on cv. Marmande leaves. Leaves were sprayed with a 50 μM peptide solution 48 h before point inoculating *B. cinerea* conidia. In a first test, both treatments and infections were performed on the abaxial surface of detached leaves. The peptides Pep 4, Pep 4r, and Pep K9 turned out to reduce significantly disease severity compared to control, as determined by necrotic lesion measurements (Figure 1A) and no efficacy increase occurred when leaves were treated with 100 μM peptide solutions (Supplementary Figure S2). Neither 50 nor 100 μM spray treatments caused visible phytotoxic effects on leaves (data not shown), and 50 μM was selected as a suitable concentration for the following assays.

**Figure 2:**
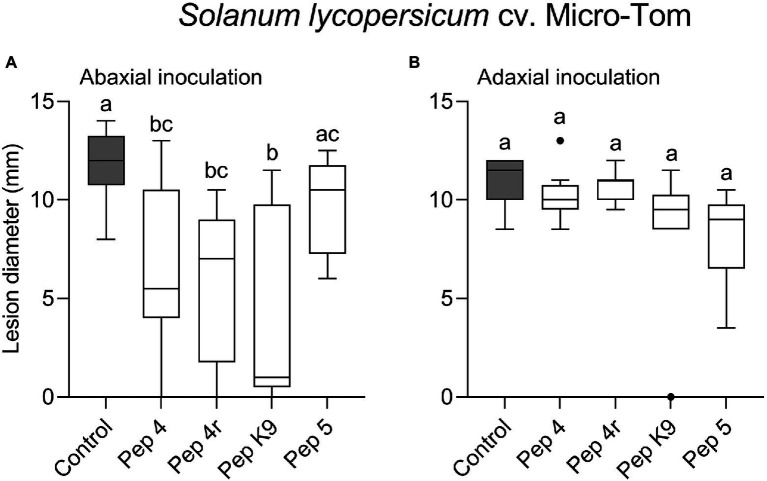
Preventive efficacy of selected Trichogin GA IV-derived peptides against *Botrytis cinerea* infection on *Solanum lycopersicum* cv. Micro-Tom leaves. Treatments were performed by spraying the lower (abaxial) surface with 50 μM peptide solution in water. The pathogen was inoculated 48 h later by drop inoculation with a conidial suspension of *B. cinerea*. Conidia were applied on the abaxial (peptide-treated; **A**) or adaxial (untreated; **B**) leaf surface. Lesion diameters were measured 3 days after infection. Control leaves were treated with water. Values from nine biological replicates are reported as Tukey boxplots. Data were analyzed by one-way ANOVA with Tukey–Kramer post-test (*p* < 0.05). Statistically significant differences are shown by different letters. The experiment in **(A)** was repeated with similar results.

**Figure 3:**
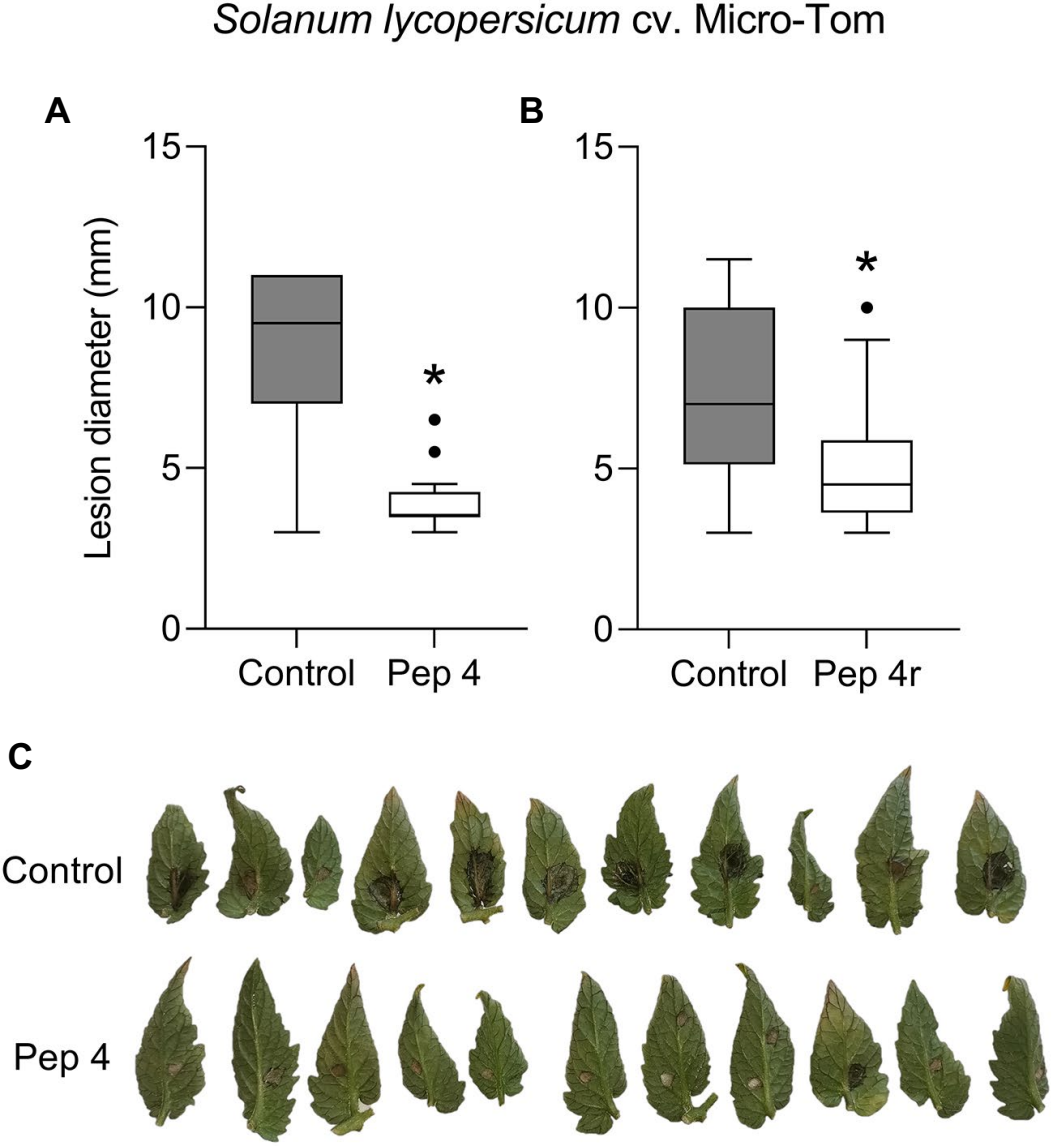
Trichogin GA IV-derived peptides Pep 4 **(A,C)** and Pep 4r **(B)** protect tomato from *Botrytis cinerea* infection up to 5 days after treatment. *Solanum lycopersicum* cv. Micro-Tom leaves were treated on the lower (abaxial) surface by spraying 50 μM peptide solution in water, and drop inoculated 120 h later with a conidial suspension of *B. cinerea*. Conidia were applied on the abaxial (peptide-treated) leaf surface. Lesion diameters were measured 3 days after infection. Control, leaves treated with water. Values from 11 to 13 biological replicates **(A)** or 12–16 biological replicates **(B)** are reported as Tukey boxplots. Data were analyzed by *t*-test (*p* < 0.05). Statistically significant differences are shown by asterisk. Representative pictures concerning Pep 4 protection from *B. cinerea* infection are shown **(C)**.

To get clues on the ability of the peptides to act as penetrating fungicides or induce localized resistance, the peptides were sprayed on the abaxial leaf surface and the pathogen was inoculated 48 h later on the opposite (adaxial) untreated leaf surface. In this case, the treatments were not effective in restricting lesion development compared to control (Figure 1B). The surfactant Silwet L-77 AG, which was used as an adjuvant in this experiment, did not interfere with fungal infection compared to the untreated control (Figure 1B). The protective effect of peptide treatment was also tested on a different tomato cultivar (Micro-Tom; Figure 2). Again, while Pep 4, Pep 4r, and Pep K9 turned out to be effective as protectants against *B. cinerea* (Figure 2A), the peptides did not reduce significantly disease progression when the pathogen was being inoculated on the opposite, untreated leaf surface (Figure 2B). The duration of the protection was investigated by treating leaves with Pep 4 and Pep 4r and inoculating the fungus 5 days later. As shown in Figure 3, the peptides were still effective in reducing lesion diameters 5 days after the peptide treatments.

**Figure 4:**
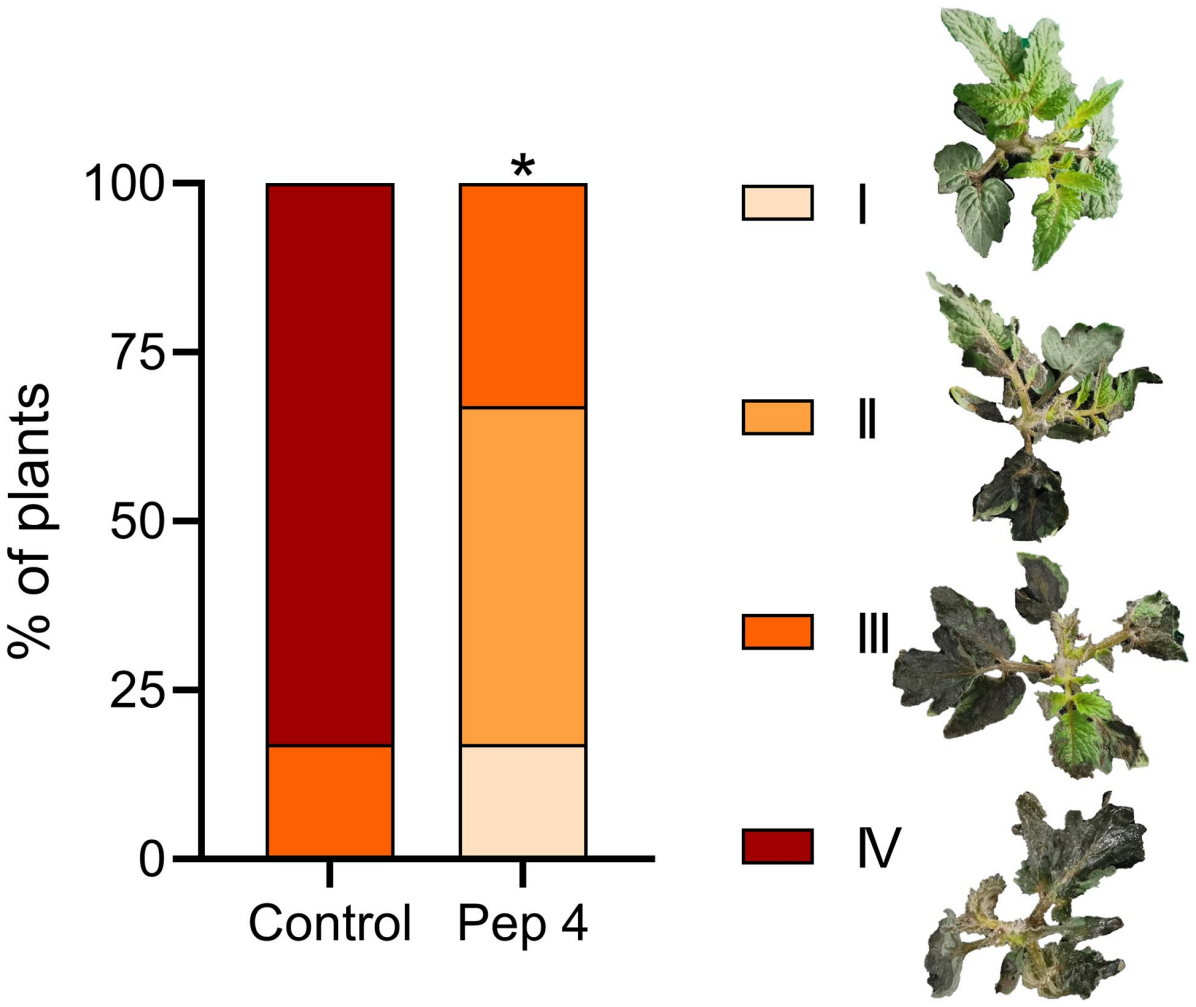
Pep 4 protects tomato plants from *Botrytis cinerea* infection. Two-week-old *Solanum lycopersicum* cv. Micro-Tom plants were sprayed on the upper (adaxial) leaf surfaces with 50 μM peptide solution in 0.01% v/v of Silwet L-77 AG, and spray inoculated on the same surface 48 h later with a conidial suspension of *B. cinerea*. Disease severity was evaluated by assigning plants to different classes, ranging from I (no or point limited necrosis) to IV (extensive necrosis and plant collapse) according to the representative examples. Statistically significant differences in class distribution were analyzed by Fisher’s exact test compared to control (water containing 0.01% v/v of Silwet L-77 AG). ^*^p < 0.05 (*n* = 6).

Finally, to ascertain whether the protective effect detected on detached leaves occurred on whole tomato plants, 2-week-old Micro-Tom plants were sprayed with 50 μM Pep 4 containing 0.01% Silwet L-77 AG to promote leaf adhesion, and inoculated 48 h later by spraying *B. cinerea* conidia. As visible in Figure 4, Pep 4-treated plants displayed reduced leaf necrotic symptoms in comparison to control plants.

### Trichogin GA IV-Derived Peptides Do Not Show Curative Efficacy

**Figure 5:**
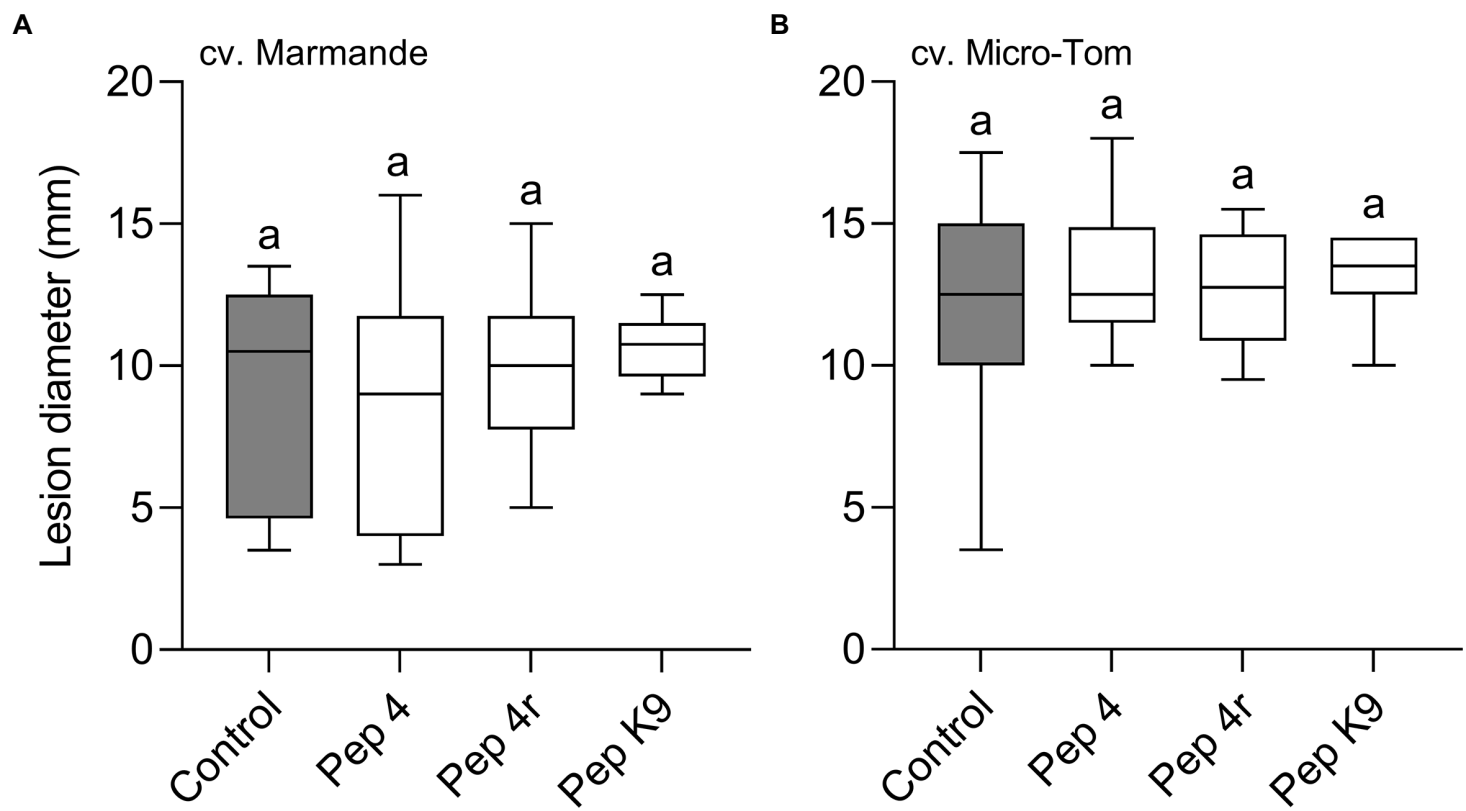
Trichogin GA IV-derived peptides do not show curative efficacy against *Botrytis cinerea* infection. The leaf abaxial surface of *Solanum lycopersicum* cv. Marmande **(A)** or Micro-Tom **(B)** was first drop inoculated with a conidial suspension of *B. cinerea*, and, 24 h later, was sprayed with 50 μM peptide solution in water containing 0.01% v/v of Silwet L-77 AG. Lesion diameters were measured 3 days after pathogen inoculation. Control leaves were treated with water containing 0.01% v/v of Silwet L-77. Values from 12 to 13 **(A)** or 13 to 15 **(B)** biological replicates are reported as Tukey boxplots. Data were analyzed by one-way ANOVA with Tukey–Kramer post-test (*p* < 0.05). Statistically significant differences are shown by different letters.

Curative efficacy against *B. cinerea* infection was investigated on both tomato cultivars, Marmande and Micro-Tom. Leaves were treated with the peptides 24 h after point inoculation of *B. cinerea* conidia, i.e., when infection symptoms beneath the inoculation droplet were already visible (not shown). Pep 4, Pep 4r, and Pep K9 at 50 μM with 0.01% Silwet L-77 AG were ineffective in reducing disease severity compared to control on either cultivar (Figures 5A,B), indicating that Trichogin GA IV-derived peptides do not possess any curative efficacy against *B. cinerea* infections.

### Induction of ROS Production

**Figure 6:**
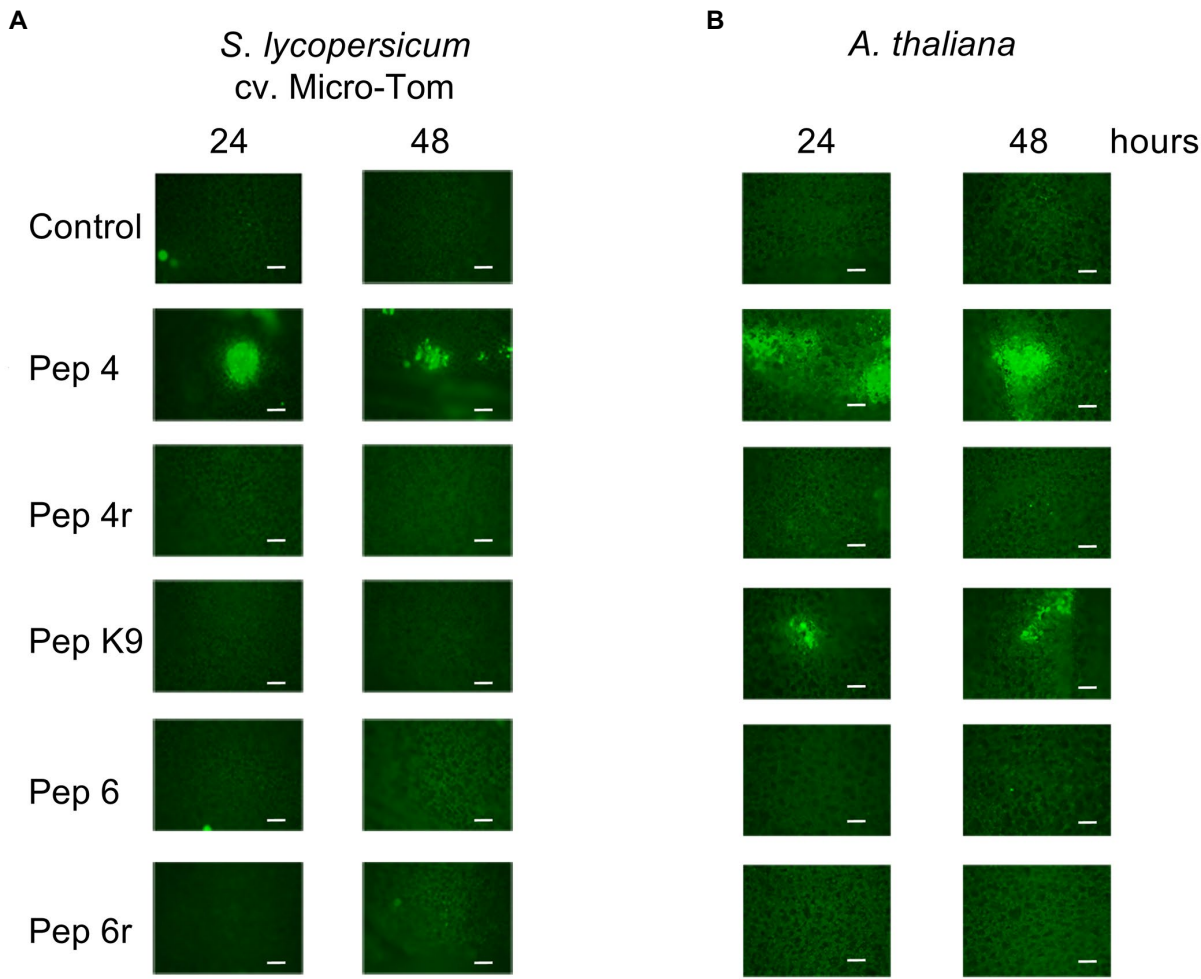
Reactive oxygen species (ROS) detection on *Solanum lycopersicum* cv. Micro-Tom **(A)** or *Arabidopsis thaliana* Col-0 **(B)** leaves. Leaves were treated with droplets containing 50 μM Trichogin GA IV-derived peptides for 24 and 48 h, and subsequently stained with the fluorescent probe 2′,7′-dichlorodihydrofluorescein diacetate (H_2_DCFDA) to visualize ROS production *in situ*. A representative picture per treatment is shown. Scale bars: 200 μm.

To understand more about their mode of action, peptides Pep 4, Pep 4r, and Pep K9 (all showing efficacy against *B. cinerea*), and Pep 6 and Pep 6r for comparison, were applied to healthy Micro-Tom and *A. thaliana* leaves. ROS were determined by fluorescence microscopy with the H_2_DCFDA probe. As visible in Figure 6A, only Pep 4-treated leaves showed ROS production on tomato leaves after 24 and 48 h. Fluorescent-stained ROS were mostly confined beneath the point of droplet application with no apparent spread to the surrounding zones. A similar result was obtained on *A. thaliana* leaves, although in that case both Pep 4 and Pep K9 induced ROS production on leaves (Figure 6B).

### Impact of Peptide Treatment on Plant Defense Gene Expression

The induction of ROS production prompted us to investigate the ability of Pep 4 to prime defenses. To do so, a gene expression analysis was designed. The following defense-related genes were selected: the ethylene (ET) biosynthesis gene *1-aminocyclopropane-1-carboxylate oxidase 1 (ACO1)*, the jasmonic acid (JA)-related gene *Lipoxygenase A (Lox1.1)*, the salicylic acid (SA)-marker gene *Pathogenesis-related protein 1 (PR1)*, and some defense genes known to be affected during *B. cinerea* infection in tomato, such as *Pathogenesis-related genes transcriptional activator 5 (PTI5)*, *Polygalacturonase-inhibiting protein (PGIP)*, *Proteinase inhibitor II (PIN2)*, and *Beta-1,3-glucanase A* (GluA; Harel et al., 2014; Sun et al., 2017; Gupta et al., 2021). Gene expression was analyzed at three different time points in order to investigate whether Pep 4 could directly induce defenses ahead of infection (48 hpt), or rather prime them for earlier/stronger induction during infection (6 and 24 hpi; Mauch-Mani et al., 2017). At each time point, control and mock-inoculated plants were collected for RNA extraction. Before collecting 24 hpi samples, the effectiveness of the treatment was verified and confirmed (Supplementary Figure S3).

**Figure 7:**
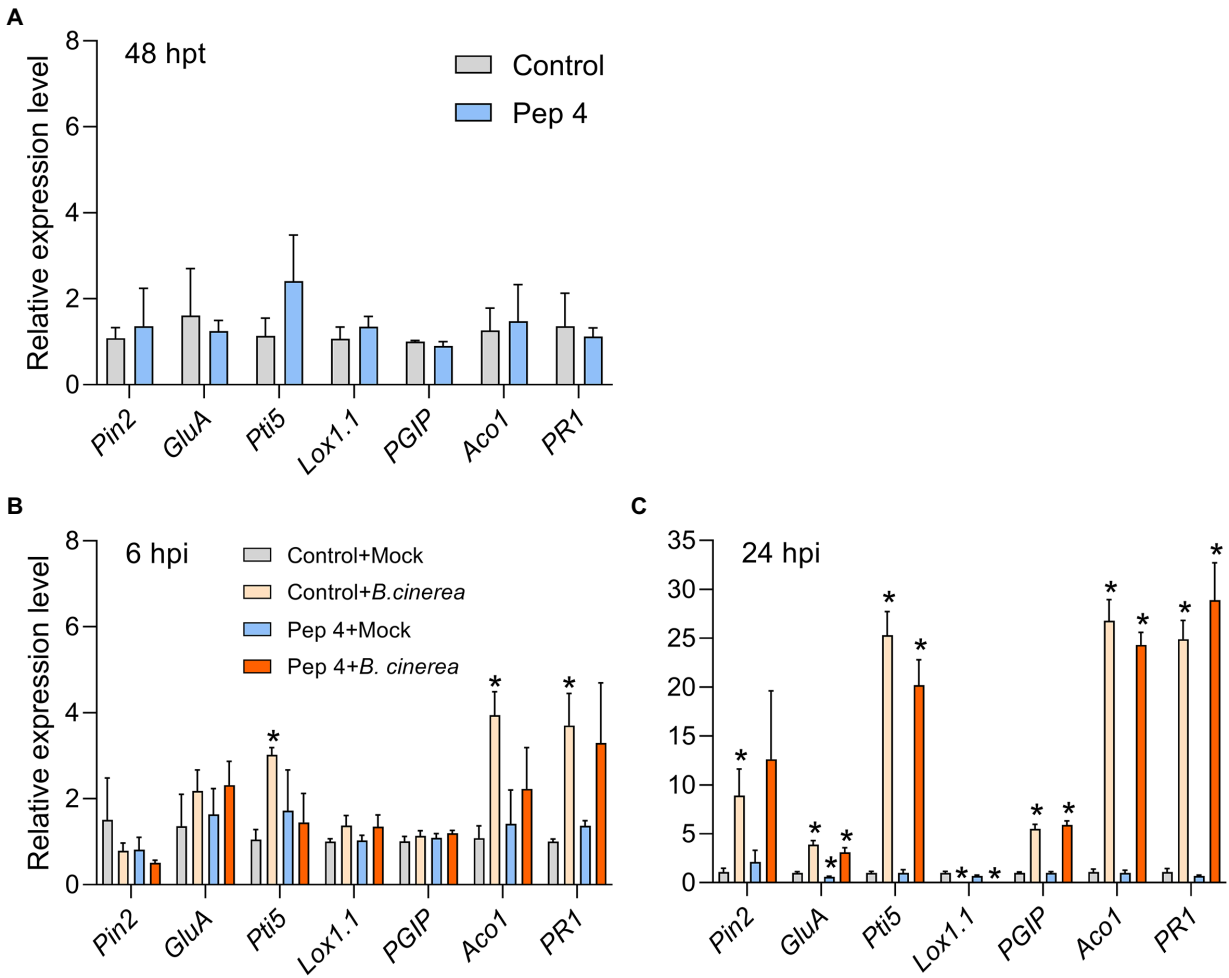
Expression of defense genes in *Solanum lycopersicum* cv. Micro-Tom plants treated with Pep 4 **(A)** and subsequently infected with *Botrytis cinerea* **(B,C)**. Peptide treatments were performed by spraying 50 μM Pep 4 in water containing 0.01% v/v Silwet L-77 AG. Pathogen inoculations were performed by spraying a conidial suspension of *B. cinerea*. Hpt, hours post treatment; hpi, hours post infection. Control, water containing 0.01% v/v Silwet L-77 AG. Mock inoculations were performed by spraying the fungal culture medium potato dextrose broth. Genes analyzed: *1-aminocyclopropane-1-carboxylate oxidase 1*(*ACO1*), *Lipoxygenase A* (*Lox1.1*), *Pathogenesis-related protein 1* (PR1), *Pathogenesis-related genes transcriptional activator 5* (*PTI5*), *Polygalacturonase inhibitor protein* (*PGIP*), *Proteinase inhibitor II* (*PIN2*), and *Beta-1,3-glucanase A* (*GluA*)*. Actin-7* was used as the endogenous reference gene for normalization. Control **(A)** or control + mock **(B,C)** were used as the calibrator sample (grey bars) for calculations. Average fold change values from three biological replicates (different plants) and two technical replicates ±SEM are shown. Statistical analysis was performed by *t*-test by comparing to the calibrator sample. Significant differences are marked by asterisk (*p* < 0.05).

Pep 4 did not significantly upregulate defense genes at 48 h post treatment (Figure 7A). The same held true at 6 and 24 hpi in mock-treated plants (i.e., 54 and 72 h after initial Pep 4 treatment; Figures 7B,C). The only exception was represented by a moderate down-regulation detectable for *GluA* in Pep 4 + mock plants after 24 hpi (i.e., 72 h after initial Pep 4 treatment; Figure 7C). In contrast, *B. cinerea* infection markedly altered gene expression: at 6 hpi, mRNA levels for *Pti5*, *ACO1*, and *PR1* genes were significantly higher (≥3-fold) in infected plants not treated with Pep 4 (Figure 7B). At 24 hpi, the same three genes turned out to be strongly up-regulated (>20-fold; Figure 7C), and all the genes analyzed were modulated: *PIN2* was significantly upregulated only in control + infection plants; *GluA*, *Pti5*, *PGIP*, *ACO1*, and *PR1* were upregulated in both control + infection and Pep 4 + infection plants, and their relative expression reached a similar fold-change value in control + infection and Pep 4 + infection; *Lox1.1* was instead strongly down-regulated in both control + infection and Pep 4 + infection plants (>50-fold; Figure 7C). Overall, these results suggested that Pep 4 treatments did not significantly induce defense gene expression nor did they enhance the defense response of the plant during infection.

## Discussion

Building on our previous work (De Zotti et al., 2020), we studied here the plant protection ability of six Trichogin GA IV-derived peptides characterized by one to three substitutions in the native Trichogin GA IV sequence (Table 1): Gly to Lys substitutions occurred in all the peptide analogs (Lys is a cationic residue that gives hydrophilicity to the peptide sequence); Gly to α-aminoisobutyric acid (Aib) substitutions were present in Pep 6 and Pep 6r (Aib is a strong helix-inducer; De Zotti et al., 2012b); leucinol (Lol) to leucine amide substitutions were present in Pep 4r and Pep 6r (the introduction of an additional hydrogen bond strengthens the helix). By replacing the Gly residue with Lys at position 9 (Pep K9), we aimed to evaluate the enhancement of the peptide hydrophilicity against the helix stabilization effect.

In our previous study, a few Trichogin GA IV-derived peptides rationally modified to acquire water-solubility showed toxic activity against plant pathogens (De Zotti et al., 2020). In particular, we reported Pep 4 as one of the most effective molecules in inhibiting germination and growth of *B. cinerea in vitro*, and restricting *B. cinerea* infections on bean and grape tissues (De Zotti et al., 2020).

In the present study, by using tomato and *Arabidopsis* plants, we investigated the mode of action in planta of six Trichogin GA IV-derived peptides, with special attention to Pep 4.

We found that Pep 4 and Pep 4r (De Zotti et al., 2020), as well as Pep K9 (designed in the present study), were effective in restricting *B. cinerea* infections on tomato leaves. These peptide analogs were effective on both the tomato cultivars tested, namely Marmande and Micro-Tom (Figures 1, 2), whereas Pep 5, Pep 6, and Pep 6r did not show protection capacity. This first result suggests peptide hydrophilicity, rather than helix stability, as the major determinant of their efficacy *in planta* (Table 1).

The treatments were effective only when the peptides were sprayed before the application of *B. cinerea* conidia on leaves, whereas no protection was detectable when the peptides were sprayed on ongoing infections (Figure 5). Therefore, we may conclude that Trichogin GA IV-derived peptaibols possess preventive but not curative efficacy against *B. cinerea* infections.

Although very limited investigations have been carried out up to date, some peptaibols have been found to act as defense elicitors and resistance inducers in plants. Alamethicin was reported, for instance, to induce the synthesis of defense hormones (Engelberth et al., 2000), the emission of volatile organic compounds (Bruinsma et al., 2009), and lead to programmed cell death and callose deposition (Rippa et al., 2010). Two synthetic peptaibols, TvBI and TvBII from *T. virens* Gv29-8, could instead induce systemic resistance against *Pseudomonas syringae* pv. *lachrymans* in *Cucumis sativus* plants (Viterbo et al., 2007). Trichokonin from *T. pseudokoningii* SMF2 was able to induce ROS production and resistance against tobacco mosaic virus in tobacco (Luo et al., 2010) and *Pectobacterium carotovorum* subsp. *carotovorum* (*Pcc*) in Chinese cabbage (Li et al., 2014), respectively. Importantly, *N*-1-octanoylated, C-terminal methyl ester analogs of Trichogin GA IV were reported to induce *PDF1.2* and *PR2* defense gene expression in *Arabidopsis* (Rippa et al., 2010). For these reasons, we hypothesized that the preventive efficacy of our synthetic analogs could be due to a dual activity when sprayed on plants: antimicrobial (toxic) against the fungus, and resistance-inducing on the plant defense system.

The ROS results (Figure 6), especially concerning Pep 4, prompted us to investigate, by gene expression analysis, the resistance-inducing hypothesis. The ET biosynthetic gene *ACO1*, the JA-related gene *Lox1.1*, and the SA-marker gene *PR1* were selected for their involvement in hormone-signaling pathways and their reported modulation during *B. cinerea*-tomato interaction: upregulation of *PR1* and *ACO1* genes, and downregulation of the *Lox1.1* gene have been reported (Harel et al., 2014; Sun et al., 2017; Gupta et al., 2021). Importantly, SA-dependent defenses are known to positively regulate basal resistance against *B. cinerea* in tomato (Achuo et al., 2004). Other defense genes known to be upregulated during *B. cinerea*-tomato interaction were *PTI5*, *PGIP*, *PIN2*, and *GluA* (Harel et al., 2014; Sun et al., 2017; Gupta et al., 2021). Some of these, such as *PGIP*, *PIN2*, and *GluA*, encode for enzymes that may play a direct antifungal role: polygalacturonase-inhibiting proteins (PGIPs) are capable of inhibiting fungal endopolygalacturonases, thereby limiting plant tissue colonization (De Lorenzo et al., 2001); proteinase inhibitors may inhibit extracellular proteases secreted by *B. cinerea* and contribute to plant resistance (Turrà et al., 2020); beta-1,3-glucanases can degrade fungal cell wall glucans (van Loon et al., 2006).

However, the gene expression analysis did not show any considerable induction of defense genes by Pep 4 in a period of 48–72 h post treatment (Figure 7). The presence of defense priming could be similarly excluded since Pep 4 did not appear to prime defense genes for a quicker/stronger activation during infection with *B. cinerea* (Figures 7B,C). On the contrary, the gene expression level indicated that the plant response was delayed in Pep 4-treated plants, as visible at 6 hpi (Figure 7B), as the probable effect of the antimicrobial activity exerted by Pep 4 which delayed/reduced the infection level. A lack of resistance-inducing/priming ability was consistent with the lack of translaminar activity observed on leaves (Figures 1B, 2B). The ability to prime defenses and induce resistance, if present, would have indeed probably allowed highlighting an efficacy on both treated and untreated leaf surfaces, evidence that we were able to exclude.

In conclusion, with this study, it seems possible to indicate the antimicrobial effect as the only mechanism behind the plant protection ability exerted by Trichogin GA IV-derived peptides. The antimicrobial effect shown on leaves likely affected conidial germination, in a way similar to what we reported *in vitro* (De Zotti et al., 2020; Sella et al., 2021). Since *B. cinerea* hyphae are similarly susceptible to Pep 4 treatment (unpublished results), we hypothesize that the peptides cannot overcome the leaf cuticle to target the infectious hyphae in the leaf mesophyll.

Synthetic analogs of Trichogin GA IV have been previously demonstrated to retain the native ability to interact with membranes (De Zotti et al., 2012a,b), therefore their antimicrobial effect is very probably due to their membrane activity (Sella et al., 2021). This activity may also be responsible for the induction of plant ROS production observed after leaf treatment with Pep 4 and Pep K9. However, the gene expression analysis did not highlight considerable modulation of defense genes by Pep 4. *PR1* gene modulation, for instance, which is known to occur at late time points after several biotic or abiotic stresses (Akbudak et al., 2020; Sewelam et al., 2021), was not altered by Pep 4 treatment in a 48–72 h period, indicating a limited stress occurrence. Therefore, the ROS result combined with the gene expression analysis suggest a transient and limited impact of Pep 4 on the plant defense system, not resulting in the activation of defense priming.

Further studies will elucidate whether, in the long term, repeated applications of Trichogin GA IV-derived peptides, despite their limited plant activity, may impact plant fitness and growth. The analog Pep 4r, showing high efficacy against infection and lack of ROS elicitation, could represent the best choice for future applications.

## Data Availability Statement

The original contributions presented in the study are included in the article/Supplementary Material; further inquiries can be directed to the corresponding author.

## Author Contributions

IB, LS, and FF conceived the present study. MDZ designed and synthesized Trichogin GA IV-derived peptides. IB and LS designed the experiments on plants. IB, SL, and RB carried out the experiments on plants. IB analyzed data and wrote the manuscript. All the authors provided their feedback during writing. All authors contributed to the article and approved the submitted version.

## Funding

This research was funded by the Italian Ministry for University and Research, PRIN 20173LBZM2. MDZ is grateful to the University of Padova (Italy) for funding (grant number: P-DiSC#04BIRD2019-UNIPD) and to the Italian Ministry for the Economic Development, MISE (grant number: PoC@Unipd–CUP 96I20000120004; Project: ECOPEP).

## Conflict of Interest

The authors declare that the research was conducted in the absence of any commercial or financial relationships that could be construed as a potential conflict of interest.

## Publisher’s Note

All claims expressed in this article are solely those of the authors and do not necessarily represent those of their affiliated organizations, or those of the publisher, the editors and the reviewers. Any product that may be evaluated in this article, or claim that may be made by its manufacturer, is not guaranteed or endorsed by the publisher.
