# Supplementary material for: Water-Soluble Trichogin GA IV-Derived Peptaibols Protect Tomato Plants From Botrytis cinerea Infection With Limited Impact on Plant Defenses
Source: Front Plant Sci. 2022 May 19;13:881961. doi: 10.3389/fpls.2022.881961 (PMC9161086; doi:10.3389/fpls.2022.881961)
Supplement: Supplementary file 1 [file Data_Sheet_1.docx]

Supplementary Material

**Supplementary Table 1.** Genes analyzed and primer sequences used for RT-qPCR.

| **Gene name** | **Locus** | **Forward primer (5’-3’)** | **Reverse primer (5’-3’)** |
| --- | --- | --- | --- |
| *Proteinase inhibitor II (PIN2)* | NM_001247698.1 | TGCCAAGGCTTGTACTAGAGAATG | CTTATAGCCTGAGCAACAATTGATG |
| *Beta-1,3-glucanase A (GluA)* | NM_001247869.2 | ACTTTTCAAGCAACAAGGGCTAA | GAATCCACAAGGGCATCGAA |
| *Pathogenesis-related protein 1 (PR1)* | NM_001247429.1 | AACCTAGCTGCCGCTTTCC | TCTCATCAACCCACATCTTCACA |
| *1-aminocyclopropane-1-carboxylate oxidase 1 (ACO1)* | NM_001247095.2 | GAGCCAAGATTTGAAGCAATGA | GAATTGGGATCTAAGCACTTGCA |
| *Pathogenesis-related genes transcriptional activator (PTI5)* | NM_001247058.2 | GCGATTCGGCTAGACATGGT | GCAGCTTCTTCAGCAGTTTCG |
| *Lipoxygenase A (Lox1.1)* | NM_001247927.2 | GTGGAAGGAAGTCCGAGAAGTG | TGGGACGATTTGGGAGGTAA |
| *Polygalacturonase inhibitor protein (PGIP)* | NM_001330726 | CCAACCGGATAAATGCTCTCA | CGGAATTTGGCCGGAGATAT |
| *Actin-7* | NM_001308447.1 | CGTACAACTGGTATTGTGTTGG | CGGTGAGGATCTTCATCAGGT |
| *Ubiquitin* | NM_001345879.1 | CAAGACATTGACAGGGAAGAC | CCACCTCTAAGGCGAAGAAC |
| *Tubulin beta chain* | XM_004240343 | TCTCATTCCTTTCCCCCGTCTCC | CATCTGCTCATCAACTTCCTTGGTGC |
| *Glyceraldehyde-3-phosphate dehydrogenase A* | XM_004236801 | CTGCTCTCTCAGTAGCCAACAC | CTTCCTCCAATAGCAGAGGTTT |

**Supplementary Figure 1**. Transcriptional stability of four different candidate endogenous reference genes (*Actin-7*, *Ubiquitin*, *Tubulin beta chain* and *Glyceraldehyde-3-phosphate dehydrogenase A - GAPDH*) across ten different cDNA samples representative of each experimental condition.

| **Sample** | **Treatment** | ***Actin***  (Ct values) | ***Ubiquitin***  (Ct values) | ***Tubulin***  (Ct values) | ***GAPDH***  (Ct values) |
| --- | --- | --- | --- | --- | --- |
| 1 | Control, 48hpt | 20.4033 | 22.17011 | 21.69557 | 15.67875 |
| 4 | Pep4, 48hpt | 20.50684 | 22.19052 | 21.91979 | 15.56273 |
| 7 | Control+mock, 6hpi | 20.54813 | 22.85227 | 25.20161 | 16.84517 |
| 10 | Control+Inf., 6hpi | 20.14689 | 22.39856 | 24.89786 | 16.37898 |
| 13 | Pep4+mock, 6hpi | 20.88157 | 23.07317 | 25.17737 | 16.81327 |
| 16 | Pep4+Inf., 6hpi | 19.81329 | 22.25773 | 24.72934 | 16.44251 |
| 20 | Control+mock, 24hpi | 20.28778 | 22.02772 | 25.09817 | 16.40279 |
| 22 | Control+Inf., 24hpi | 19.91173 | 20.26717 | 26.53268 | 18.90939 |
| 25 | Pep4+mock, 24hpi | 20.17453 | 21.9249 | 25.22546 | 16.15486 |
| 28 | Pep4+Inf., 24hpi | 20.26791 | 20.51674 | 26.47465 | 18.9706 |
|  |  |  |  |  |  |
|  | Average Ct value | 20.29 | 21.97 | 24.70 | 16.82 |
|  | SD | 0.31 | 0.90 | 1.64 | 1.19 |

**Supplementary Figure 2.** Efficacy of Trichogin-derived peptides at 100 µM. Treatments were performed on *Solanum lycopersicum* cv. Marmande leaves 48 h before *B. cinerea* B05.10 inoculation. Treatments and pathogen inoculations were performed on the same (abaxial) leaf surface. Data are the mean ± SEM (n=12). Statistical analysis was performed by unpaired *t*-test by comparing 100 µM data to 50 µM data from Figure 1, and considered significant at *P*<0.05. n.s., not significant.

n.s

n.s

n.s

n.s

n.s

n.s

**Supplementary Figure 3.** Disease severity 24 h after spray inoculation of *B. cinerea* conidia on plants used for RNA extraction. Pep 4 treatments (50 µM + Silwet L77 AG 0.01%) were performed on 2-weeks-old plants 48 h before *B. cinerea* B05.10 spray inoculation. Peptide treatment and pathogen inoculation were performed on the same (adaxial) leaf surface. Disease severity was quantified as the number of necrotic spots visible on the adaxial leaf surfaces 24 h after pathogen inoculation, just before freezing in liquid nitrogen for RNA extraction. Data are shown as mean ± SEM (n=3 plants). Statistical analysis was performed by unpaired *t*-test. *, statistically significant at *P*<0.05.

*
